# A Comprehensive Perspective on Febrile Seizures in Children: A Prospective Cohort Study with Evaluation of Clinical, Laboratory, and Genetic Features

**DOI:** 10.3390/jcm14227918

**Published:** 2025-11-08

**Authors:** Gülşen Yalçın, Ruken Yıldırım, Edip Unal, Dilek Cebeci, Atilla Ersen, Berk Özyılmaz, Selahattin Tekeş, Murat Anıl, Aylin Gürbay

**Affiliations:** 1 Buca Seyfi Demirsoy Training and Research Hospital, İzmir Democracy University, İzmir 35390, Turkey; drgyalcin@gmail.com (G.Y.); atillaersen@hotmail.com (A.E.); 2 Faculty of Pharmacy, Department of Pharmaceutical Toxicology, Hacettepe University, Ankara 06100, Turkey; 3 Diyarbakır Children’s Hospital, Diyarbakır 21010, Turkey; rukmay21@hotmail.com; 4 Department of Pediatrics, Dicle University Faculty of Medicine, Diyarbakır 21280, Turkey; edip76@yahoo.com; 5 Gazi Yaşargil Training and Research Hospital, Diyarbakır 21070, Turkey; dilekcebeci@yahoo.com; 6 İzmir City Hospital, University of Health Sciences, İzmir 35530, Turkey; drberk@gmail.com; 7 Department of Medical Genetics, Dicle University Faculty of Medicine, Diyarbakır 34000, Turkey; selahattintekes@gmail.com; 8 Faculty of Medicine, Department of Pediatrics, İzmir Democracy University, İzmir 35160, Turkey; muratanil1969@gmail.com

**Keywords:** febrile seizures, complex febrile seizures, biochemical markers, genetic variants, sodium, vitamin D, zinc, ferritin

## Abstract

**Background:** Febrile seizures (FS) are the most common seizures in childhood, yet their clinical, biochemical, and genetic risk factors are still being clarified. This study aimed to provide a comprehensive evaluation of FS from clinical, laboratory, and genetic perspectives. **Methods:** In this prospective cohort study, 124 children aged 6 months to 5 years presenting with FS and 93 febrile controls without seizures were evaluated. Clinical features, laboratory parameters (including trace elements and hormonal markers), and genetic analysis using a 37-gene epilepsy panel were assessed. Multivariate logistic regression analysis was performed to identify independent predictors of FS, complex FS, and recurrent seizures. **Results:** Children with FS had significantly lower serum sodium, vitamin D, and zinc levels compared to controls. Multivariate analysis identified low sodium and low vitamin D levels as independent risk factors for FS. In the subgroup analysis, lower sodium and vitamin D levels and elevated ferritin levels were independently associated with complex FS. Lower serum zinc levels were significantly associated with seizure recurrence. Genetic analyses revealed pathogenic or likely pathogenic variants in 15.7% of patients with FS, predominantly involving SCN1A and PCDH19 genes. Patients with pathogenic variants also exhibited significantly lower levels of zinc, and selenium compared to genetically negative patients. **Conclusions:** This study highlights that metabolic disturbances, particularly involving sodium, vitamin D, and zinc, play a crucial role in FS occurrence, complexity, and recurrence. Ferritin may serve as a more sensitive indicator of inflammatory processes influencing seizure severity compared to CRP. Furthermore, genetic predispositions, especially SCN1A and PCDH19 variants, may underlie susceptibility in a subset of children. Routine evaluation of biochemical markers and consideration of genetic testing in selected cases may enhance individualized management strategies for FS.

## 1. Introduction

Febrile Seizure (FS) is the most common type of seizure disorder in childhood, affecting approximately 2–5% of children aged six to 60 months, and are generally considered benign [1]. The prevalence of FS varies geographically, with a reported rate of 5.5–9.7% in Turkey [2]. The pathogenesis of FS involves multiple factors and remains debatable [3]. Main risk factors include a family history of FS, viral infections, prolonged neonatal intensive care unit stays, maternal smoking during pregnancy, and deficiencies in vitamins and trace elements such as pyridoxine, iron, selenium, and zinc [3,4]. Indeed, most FSs are harmless and benign, however, sudden unexplained deaths and an increased risk of psychiatric disorders have occasionally been reported [5,6]. Predicting the prognosis of FS can sometimes be challenging, necessitating preparedness regarding the potential causes, risk factors, and management principles. Reliable biomarkers for diagnostic evidence are lacking, highlighting the need for further research. Therefore, reviewing and interpreting existing FS data and their potential pathophysiological causes can be beneficial.

Over the past decade, considerable progress has been achieved in the identification of susceptibility genes and related biochemical factors associated with FSs. Nevertheless, the complex interaction between genetic predisposition and metabolic or inflammatory disturbances remains incompletely understood. Understanding these relationships may provide novel insights into the mechanisms that influence seizure threshold and recurrence risk in children with FSs.

Given this background, the objective of this study was to identify the potential risk factors (clinical, biochemical and genetic) of FSs in children aged six months to five years who are admitted to our pediatric emergency department.

## 2. Materials and Methods

### 2.1. Study Design

This is a prospective cohort study. After obtaining approval from the independent ethics committee (11 September 2020/542), the study was conducted between 1 October 2020, and 31 January 2023. This work was performed in accordance with the Declaration of Helsinki and followed the principles of good clinical practice. This study was conducted at a single tertiary pediatric emergency department at Diyarbakır Children’s Hospital, Diyarbakır, Turkey.

The study group included 124 children aged six months to five years who were admitted to the pediatric emergency department with FS and control group consisted of randomly selected 93 cases with complaints of fever between 1 October 2020, and 31 January 2023. All of the cases and controls were recruited prospectively.

### 2.2. Study Patients

Each patient was diagnosed with FS by a pediatric emergency specialist according to the American Academy of Pediatrics criteria. Children aged six months to five years without neurological history and who experienced one or more febrile seizures were included in the study. FS were categorized as simple or complex. Simple febrile seizure (SFS) was defined as a generalized tonic–clonic seizure lasting less than 15 min and occurring only once within a 24 h period in a febrile child without central nervous system (CNS) infection or significant metabolic disorder. Complex febrile seizure (CFS) was defined as a seizure lasting more than 15 min, having a focal onset, or occurring more than once within a 24 h period.

The control group consisted of neurologically normal children who were admitted to the pediatric emergency department with febrile illness, but without seizures, matched for age, sex, and socio-epidemiological characteristics. Control subjects were randomly selected among febrile patients who visited the pediatric emergency department. Exclusion criteria included the presence of chronic neurological disease, developmental delay, epilepsy, age < 6 months or >5 years, recent antibiotic use, trauma, febrile delirium, status epilepticus, breath-holding spells, febrile myoclonus, acute electrolyte imbalance, abnormal magnetic resonance imaging (MRI) findings in previous records, chronic systemic illness, previous CNS infection, history of diabetes, and poisoning.

### 2.3. Study Procedures

Upon presentation to the pediatric emergency department, detailed histories were obtained, including maternal medication use, smoking, alcohol consumption, substance use during pregnancy, illnesses, history of miscarriage or stillbirth, consanguinity between parents, birth order, developmental delays, seizure history in siblings and close relatives, mode of delivery, asphyxia history, hospital admissions, corticosteroid treatment, for >28 days stay in neonatal unit, and developmental milestones. Data were notified by members of the research team using a standard template and patients retrospective chart reviews.

Patient demographics, including age, sex, body temperature, seizure duration, seizure type, number of seizures, physical examination findings, and weight and height measurements as well as clinical laboratory analysis, were recorded. Patients were monitored, and if a seizure recurred within 24 h, they were classified as CFS.

Once stabilized, blood samples were collected for full blood count, biochemistry, and C-reactive protein (CRP), a non-specific marker of inflammation, to investigate the correlation between serum inflammatory cytokines and the severity of febrile illness. CRP level above 5 mg/dL was considered the presence of inflammation.

Initial peripheral venous blood samples were taken within an average of 30 min after presentation and before administration of corticosteroids, beta-adrenergic drugs, and/or intravenous fluids. All blood analyses were performed using the initial samples collected after obtaining informed consent from the parents or legal guardians.

Laboratory measurements included hemoglobin (Hb), hematocrit (Hct), white blood cell (WBC), and platelet (PLT) counts, mean corpuscular volume (MCV), ferritin, and iron levels, total iron-binding capacity, prolactin and cortisol levels, vitamin B12, folate, 25-hydroxy vitamin D, free T4 (FT4), TSH, sodium, potassium, chloride, serum glucose, aspartate transaminase (AST), alanine aminotransferase (ALT), urea, creatinine, CRP, calcium, magnesium, phosphorus, zinc, selenium levels, and genetic analyses.

Full blood counts were performed using the electric impedance and optical scatter methods (LH 780 device, Beckman Coulter, Brea, CA, USA). Hormone assays were performed using electrochemiluminescence immunoassay methods (Cobas E-601, Roche, Basel, Switzerland), and biochemical tests were conducted using spectrophotometry (Architect 16000, Abbott, Chicago, IL, USA). Serum selenium levels were measured with inductively coupled plasma mass spectrometry (NexIONTM 300X ICP-MS, Perkin Elmer, Waltham, MA, USA) and zinc levels were measured using atomic absorption spectroscopy (Shimadzu, Shimadzu AA-6800, Kyoto, Japan).

### 2.4. Molecular Analysis

DNA was isolated from the peripheral blood samples using the QIAamp DNA Blood Mini Kit (QIAGEN, Hilden, Germany) according to the manufacturer’s instructions. Next-generation sequencing (NGS) was performed using a custom targeted NGS panel, covering 37 genes (Table 1) associated with epilepsy and febrile seizures (CDHS-37477Z-1511) (QIAGEN, Hilden, Germany). The 37-gene panel was selected because it includes genes most frequently associated with epilepsy and febrile seizures, based on previously validated epilepsy gene panels.

Libraries were pooled and sequenced on a MiSeq platform (Illumina, San Diego, CA, USA). Data analysis was performed using Qiagen Clinical Insight (QCI), Geneticist Assistant (GA) (Soft Genetics, State College, PA, USA), and Franklin (Genoox, Palo Alto, CA, USA) bioinformatics software. Variants with a minimum coverage of 50× were analyzed.

### 2.5. Statistical Analysis

Data analysis was performed using the Statistical Package for Social Sciences (SPSS) 23.0 (IBM, Armonk, NY, USA). Categorical variables were expressed as numbers and percentages [n(%)], and continuous variables were presented as median (minimum-maximum) values. Nominal variables were compared using the Chi-square test or Fisher’s exact test, as appropriate. Parametric methods were used for normally distributed continuous variables, and the Independent Sample-*t* test was employed for comparisons between two independent groups. For non-normally distributed continuous variables, non-parametric test was used, and the Mann–Whitney U test was applied for comparisons between two independent groups. Multivariate logistic regression was performed to identify independent factors associated with three outcomes: 1. Presence of febrile seizure (patient vs. control); 2. Occurrence of complex febrile seizures; 3. Occurrence of recurrent seizures (≥2 episodes). Candidate variables for inclusion in the multivariate model were selected based on the results of univariate analyses (*p* < 0.10). The Variance Inflation Factor (VIF) was used to assess multicollinearity among predictors. Issues related to perfect separation were addressed by restricting the number of variables in the final model when necessary. All results were reported as odds ratios (ORs) with 95% confidence intervals (CIs), and a *p*-value < 0.05 was considered statistically significant.

Informed consent was obtained from the parents or legal guardians of all participants involved in the study.

## 3. Results

### 3.1. Clinical Features

This study included 124 patients with FS (mean age: 21.5 months) and 93 febrile controls without seizures (mean age: 24 months). Table 2 shows the demographic and perinatal characteristics of the children with FS and controls. There was no significant difference between the groups regarding age and gender. Medical drug use during pregnancy—defined as the use of any prescription or over-the-counter medication by the mother during gestation—was more common in the FS group (*p* = 0.03). The variable ‘Diagnosis’ represents the underlying febrile illness identified at admission and was compared between the patient and control groups. A statistically significant difference was observed between groups (*p* = 0.05).

Table 3 summarizes the biochemical findings of the groups. Serum sodium, vitamin D, and zinc levels were significantly lower in the FS group than in the control group (*p* < 0.05). Multivariate logistic regression analysis identified low sodium and low vitamin D levels as independent risk factors for FS.

### 3.2. Laboratory Parameters

The hemogram parameters (Hb, Hct, PLT, and MCV) were found significantly decreased in the study group compared to the control group. In biochemical and hormone tests, the levels of serum vitamin B12, vitamin D, sodium, potassium, calcium, and zinc were significantly decreased in the study group (*p* < 0.05) (Table 4). Multivariate logistic regression analysis demonstrated that lower serum sodium and lower vitamin D levels were independently associated with the presence of febrile seizures compared to the control group. Specifically, serum sodium (OR: 0.57; 95% CI: 0.48–0.67; *p* < 0.001) and vitamin D levels (OR: 0.96; 95% CI: 0.93–0.99; *p* = 0.014) were significantly lower in patients with febrile seizures.

As shown in Table 5, Hb, Hct, PLT, serum iron, vitamin D, sodium, zinc, and selenium levels were significantly lower in the CFS group (*p* < 0.05). Among patients with febrile seizures, multivariate analysis revealed that lower serum sodium (OR: 0.63; 95% CI: 0.48–0.83; *p* = 0.001), lower vitamin D (OR: 0.57; 95% CI: 0.43–0.75; *p* < 0.001), and higher ferritin levels (OR: 1.03; 95% CI: 1.007–1.048; *p* = 0.007) were significantly associated with CFS.

Table 6 shows a significant decrease in serum vitamin D, zinc, and selenium levels in patients with recurrent FS (*p* < 0.05). In the analysis of factors related to seizure recurrence (≥2 episodes), only lower serum zinc levels were significantly associated with an increased risk of recurrent seizures (OR: 0.98; 95% CI: 0.97–1.00; *p* = 0.011).

### 3.3. Genetic Analysis Results

Genetic epilepsy panel analysis, including 37 genes, was performed using NGS on 114 patients with FS. We considered ‘genetic positive’ patients as ‘Pathogenic (P)/Likely Pathogenic (LP) variants. Among them Pathogenic/Likely Pathogenic variants were detected in 15.7% (n = 18) of patients. Additionally, we put forth all the variant analysis results, including also Likely Benign (LB) and Variant of Uncertain Significance (VUS) reports detected in our patients (Table 7). The aim here is to provide a resource for other researchers who can detect these variants. In addition, the possibility of reaching a classification that can explain the clinic with future updated data of VUS variants, even if rare.

Variants were identified in SCN1A in 4.8% (n = 5) and PCDH19 in 2.6% (n = 3) of the patients. Other variants were found in AP4B1, ASPM, BTD, CACNA1A, CHD2, KCNJ1, LDRL, PLA2G6, SCN2A, and SCN9A (Table 7). Table 8 shows that when comparing FS patients with positive genetic test results to other FS patients, there were significant decreases in blood levels of iron, vitamin D, zinc, and selenium (*p* = 0.029, *p* < 0.001, *p* < 0.001, and *p* < 0.001, respectively). However, in multivariate logistic regression analysis, only zinc and selenium levels remained independently associated with genetic positivity. Each unit increase in serum zinc level was associated with approximately a 4% reduction in the odds of genetic positivity (OR: 0.957, 95% CI: 0.926–0.990), while each unit increase in serum selenium level was associated with about a 24% reduction in the odds (OR: 0.763, 95% CI: 0.663–0.878). Novel variants were identified in 5 patients. Patient 1 had a novel heterozygous c.263_264del mutation (p. Q88Rls*25) variant in the AIMP1 gene (ENST00000394701.4). Case 3 had a novel heterozygous c.5430_5431insAGTA (p. A1811Sfs*7) variant in the ASPM gene (NM_018136.5). Case 8 had a novel heterozygous c.755G>T (p.R252L) variant in CLN6 (NM_017882.3). Case 22 had a novel c.1707del T mutation (p. Asn570Thrfs) variant in the PCDH19 gene (NM_001184880.2). Case 34 had a *novel* heterozygous c.5790_5797dupAGATATGG (p. Ala1933fs rs746685972) variant of SCN9A. Additionally, variant of uncertain (or unknown) significance (VUS) variants were identified in 12 patients with AIMP1, CLN6, CNTNAP2 (two patients), GOSR2, IL6, KCNQ3, KCNT1, LGI1, MAGI2, MFSD8, and SCN1A (Table 7).

## 4. Discussion

### 4.1. Clinical and Biochemical Findings

Most parents perceive FS is a life-threatening and extremely dramatic event. Moreover, it is the most common seizure seen in the pediatric population during out-of-hospital and emergency department visits. Although most children with FS recover without sequelae, approximately 2–7% may later develop epilepsy, particularly those with complex or recurrent FS, underscoring the importance of identifying clinical and genetic predictors. Therefore, the concern for permanent neurologic sequelae and future epilepsy after FSs has resulted in a significant amount of research on this topic.

In our study, we aimed to evaluate this condition from multiple perspectives. We recorded histories from the prenatal period onwards and compared the clinical features and laboratory parameters of children with FS and the control group. Moreover, we hypothesized that this study is the first to investigate the relationship between genetic variants and laboratory parameters in FS.

Smoking harms not only expectant mothers, but also their unborn children. Children of mothers who smoke during pregnancy may have a higher risk for FSs. Indeed, maternal smoking during pregnancy is associated with an increased risk of FS [7]. In our study, a significant difference in maternal smoking during pregnancy was found between the FS and control groups. Our results were consistent with the existing literature, suggesting that maternal smoking during pregnancy increases the risk of FS in the offspring.

Mineral and trace element deficiencies are speculated to play critical roles in seizure risk [8]. Abnormalities in serum sodium levels can cause epileptic activity through hypo osmolarity and neuronal hyperexcitability. Relative hyponatremia during febrile illness has been indicated as a predictor of recurrent febrile seizures [9]. In our study, sodium levels were significantly lower in both the FS group compared with controls and in the CFS group compared with the SFS group (*p* < 0.001 for both comparisons). These findings support the hypothesis that hyponatremia may contribute to FS pathogenesis. Larger prospective studies are needed to clarify whether hyponatremia is a cause or a consequence of FS.

Iron deficiency anemia influences nutritional status, growth, and immunity, and all these factors may directly affect FS risk. Iron metabolism is also involved in myelination and neurotransmitter activity in the nervous system [10]. Although some studies report that iron deficiency anemia increases FS risk, others suggest a possible protective role [11]. The peak ages for FS and iron deficiency anemia overlap, prompting numerous studies to examine this association. Our study showed that Hb, Hct, MCV, and serum iron values, as well as transferrin saturation, were significantly lower in FS patients than in controls, while ferritin levels were significantly higher (*p* < 0.001) [12].

Although confounding factors such as nutritional status, infection rates, and cultural differences represent study limitation, our data support the hypothesis that iron deficiency may increase FS risk. Ferritin is an acute-phase reactant; therefore, future randomized studies should explore whether elevated ferritin reflects inflammation or altered iron metabolism in FS.

Serum prolactin and cortisol levels were found to be elevated in our patients, consistent with prior findings suggesting postictal neuroendocrine activation. These results may contribute to future studies aimed at identifying hormonal biomarkers useful in differentiating febrile seizures from other paroxysmal events.

In our study, no significant difference in thyroid hormones was found between groups, consistent with previous reports [13].

### 4.2. Vitamin D, Zinc, and Selenium

The role of vitamin D in FS remains under investigation. In our study, vitamin D levels were significantly lower in the patient group compared with controls, and lower in the CFS group than in the SFS group, consistent with Bhat et al. [14].

Similarly, serum zinc levels were significantly lower in FS patients compared with febrile controls and lower in CFS than in SFS. These findings align with the literature suggesting that low zinc levels increase FS risk [15,16]. Selenium levels, though less studied, were also reduced in CFS compared with SFS, supporting previous research [17,18].

Even though vitamin D, serum zinc, and selenium levels appear to be helpful markers of susceptibility or prognosis in FS, considerable overlap exists among individual measurements. Therefore, large-scale, prospective, randomized studies are required to verify these associations while considering confounding factors such as nutrition, infection frequency, cultural factors, and genetic background. Moreover, future studies should evaluate whether supplementation of vitamin D, zinc, or selenium could reduce FS susceptibility or recurrence risk.

### 4.3. Comparison of Simple and Complex FS

Multivariate analysis revealed that low serum sodium and vitamin D were independently associated with FS occurrence. Children with reduced sodium levels were significantly more likely to experience febrile seizure, supporting existing evidence that even mild hyponatremia can increase neuronal excitability and lower the seizure threshold. Likewise, low vitamin D deficiency known for its neuroprotective and anti-inflammatory effects was linked to a higher probability of seizure occurrence.

When comparing simple and CFS, children with complex features exhibited more pronounced biochemical disturbances. Lower sodium and vitamin D levels substantially increased the likelihood of prolonged or multiple seizures within the same febrile episode. Elevated ferritin levels also independently contributed to CFS risk, suggesting that ferritin may serve as a more sensitive indicator of seizure severity than CRP due to its combined role in inflammation, oxidative stress, and iron metabolism.

Children who experienced recurrent FS had lower serum zinc levels, indicating that marginal zinc deficiency may predispose to repeated seizure episodes. These findings collectively support the contribution of micronutrient deficiencies to FS pathogenesis.

### 4.4. Genetic Findings

Previous studies have shown that FS can be associated with genetic predisposition [19]. Polymorphisms in voltage-gated sodium channel subunits (SCN1A, SCN1B, SCN2A) and GABA(A) receptor subunits (GABRG2 and GABRD), are known to influence seizure threshold [20,21]. In our cohort, pathogenic or likely pathogenic variants were identified in several genes, most notably SCN1A (n = 5) and PCDH19 (n = 3), along with other variants in AP4B1, ASPM, BTD, CACNA1A, CHD2, KCNJ1, LDRL, PLA2G6, SCN2A and SCN9A.

The SCN1A gene encodes the neuronal sodium channel NaV1.1 and is implicated in a spectrum of seizure disorders ranging from FS and GEFS+ to Dravert syndrome [22]. In our study, pathogenic variants in SCN1A formed the largest subgroup among genetically positive cases. Although most SCN1A mutations occur de novo, the presence of a family history of FS may still indicate shared susceptibility mechanisms rather than direct inheritance. These findings highlight the clinical importance of early SCN1A testing in children with FS when a genetic predisposition is suspected.

Regarding PCDH19, our findings align with previous studies showing that variants in this gene predominantly affect females and lead to early-onset, fever-sensitive seizures accompanied by neuropsychiatric manifestations [23]. Clinicians should consider PCDH19 analysis in refractory or atypical FS presentations.

Variants in CHD2, CACNA1A, SCN2A, SCN9A, and other genes identified in our cohort have been previously linked to epileptic syndromes. The diversity of variants emphasizes that FS likely represents a complex interplay between genetic susceptibility and metabolic or inflammatory triggers. In our study, all families of patients with positive genetic tests were counseled by a genetic specialist.

It is well talked about that these trace elements interfere with the FS mechanism by aggravating the genetic variants effects. Nutrient deficiency combined with the genetic variants may lower the seizure threshold. Indeed, genetic factors play an important role in FS susceptibility with some identified syndromes; e.g., genetic epilepsy with febrile seizures plus (GEFS+). Mutations have been identified so far mostly in sodium channel subunit genes, especially SCN1A and as well as GABA receptor subunit GABRG2 and the presynaptic protein syntaxin 1B (STX1B) [24]. Actually, loss-of-function of SCN1A leads to hypoexcitability of GABAergic neurons [25]. We thought that trace elements such as zinc and selenium may involve in the etiopathogenesis of FS in this respect. Because zinc modulates GABA levels by influencing the pyridoxal kinase activity and selenium is an important nutrient in neuronal depolarization by increasing GABA levels through the glutathione peroxidase pathway [26].

Additionally, lower serum zinc and selenium levels were independently associated with genetic positivity, suggesting that micronutrient deficiencies may not only contribute to seizure susceptibility but also modulate the phenotypic expression of genetic variants. These findings may guide future translational research exploring how trace element homeostasis interacts with genotype to influence seizure outcomes.

### 4.5. Limitations

Our study was conducted in a single geographic and ethnic population, and data on socioeconomic status were not collected. Therefore, the influence of nutritional or environmental confounders cannot be fully assessed. Despite these limitations, our results provide novel insight into the combined clinical, biochemical, and genetic determinants of FS.

## 5. Conclusions

This study reinforces the concept that FS, while generally benign, can signal underlying metabolic vulnerabilities that increase the risk for more complex or recurrent seizures. Identifying and addressing modifiable biochemical abnormalities—such as correcting sodium, vitamin D, and zinc deficiencies—may offer new opportunities for risk mitigation and individualized follow-up strategies in children experiencing FSs. Moreover, significant progress has been made in understanding the biological importance of genetic variants, and we aimed to emphasize the importance of considering genetic analyses especially in suspected clinical presentations of FSs. Integrating clinical, biochemical, and genetic perspectives can enhance early risk stratification and improve personalized management strategies for affected children.

Differentiating our perspective in line with new information of clinical significance of the variants is important for a better understanding of diagnostic approaches in FSs. We herein wanted to give a wider perspective of view in FS from the perinatal period in respect to evaluation of clinical, laboratory, and genetic features. Over the past decade, the identification of responsible genes for this condition, determination of clinical phenotypes, and development of animal models have advanced significantly. Current treatments continue to focus on seizure management with conventional antiepileptic drugs; however, there remains an unmet need for targeted therapies addressing the molecular and metabolic mechanisms underlying FS.

Future research should include large-scale, multicenter, and longitudinal studies incorporating standardized neurodevelopmental, neuropsychiatric, and quality-of-life assessments to better delineate the disease spectrum and identify modifiable determinants of outcome.

## Figures and Tables

**Table 1:** Genes examined within the scope of the panel.

AP4B1	CPA6	GABRG2	KCNQ2	NPRL2	PTGS2	SCN9A
CHD2	DEPDC5	GRIN2A	KPTN	NRXN1	SCN1A	SEZ6
CHRNA4	GABBR1	HCN2	MASS1	NRXN2	SCN1B	SLC2A1
CLCN6	GABRA2	IL6	ADGRV1	PCDH19	SCN2A	SLC6A1
CNKSR2	GABRD	IMPA2	MEF2C	PRRT2	SCN8A	SPECC1L
STX1B	SYNGAP1					

**Table 2:** Distribution of demographic and clinical findings of participants.

Variables	Control (n = 93) n (%) or Mean ± SD (Minimum-Maximum) or Median (Interquartile Range)	Patient (n = 124) n (%) or Mean ± SD (Minimum-Maximum) or Median (Interquartile Range)	Total (n = 217) n (%) or Mean ± SD (Minimum-Maximum) or Median (Interquartile Range)	*p*-Value
Age (months)	24 (12–36)	21.5 (12–36)	24 (12–36)	0.517 ^a^
Gender				0.550 ^c^
Male	57 (61.3) *	71 (57.3) *	128 (59) *	
Female	36 (38.7) *	53 (42.7) *	89 (41) *	
Weight SD score	0.2 (−1.2–1.3) ***	0.2 (−1.4–1.7) ***	0.2 (−1.4–1.7) ***	0.771 ^a^
Height SD score	0.1 ± 0.07 (−1–1.3) **	0.1 ± 0.06 (−2.3–1.8) **	0.1 ± 0.6 (−2.3–1.8) **	0.863 ^b^
BMI SD score	0.1 ± 0.04 (−0.8–1.2) **	0.08 ± 0.03 (−1.8–3) **	0.08 ± 0.03 (−1.8–3) **	0.287 ^b^
Seizure Duration (minutes)	0 (0–0) ***	4 (1–20) ***	4 (1–20) ***	NA *
Seizure Type				NA *
SFS	0 (0) *	86 (69.4) *	86 (69.4) *	
CFS	0 (0) *	38 (30.6) *	38 (30.6) *	
Family History	8 (8.6) *	83 (66.9) *	91 (41.9) *	<0.001 ^c^
FS (Father)	1 (1.1) *	27 (21.8) **	28 (12.9) *	<0.001 ^c^
FS (Mother)	3 (3.2) *	7 (5.6) *	10 (4.6) *	0.521 ^c^
FS (Brother/Sister)	1 (1.1) *	21 (16.9) *	22 (10.1) *	<0.001 ^c^
FS (Uncle)	0 (0) *	7 (5.6) *	7 (3.2) *	0.021 ^c^
FS (Cousin)	3 (3.2) *	11 (8.9) *	14 (6.5) *	0.163 ^c^
FS (Aunt)	0 (0) *	6 (4.8) *	6 (2.8) *	0.039 ^c^
Epilepsy (Uncle)	0 (0) *	3 (2.4) *	3 (1.4) *	0.262 ^c^
Epilepsy (Mother)	0 (0) *	4 (3.2) *	4 (1.8) *	0.137 ^c^
Epilepsy (Brother/Sister)	0 (0) *	5 (4) *	5 (2.3) *	0.072 ^c^
Epilepsy (Aunt)	0 (0) *	4 (3.2) *	4 (1.8) *	0.137 ^c^
Affinity				0.073 ^c^
2. degree relative	9 (9.7) *	25 (20.2) *	34 (15.7) *	
3. degree relative	17 (18.3) *	15 (12.1) *	32 (14.7) *	
Smoking During Pregnancy	12 (12.9) *	46 (37.1) *	58 (26.7) *	<0.001 ^c^
Medical Drug Use During Pregnancy	2 (2.2) *	12 (9.7) *	14 (6.5) *	0.051 ^c^
Cancer	0 (0) *	1 (0.8) *	1 (0.5) *	
Diabetes Mellitus	1 (1.1) *	6 (4.8) *	7 (3.2) *	
Hypertension	1 (1.1) *	4 (3.2) *	5 (2.3) *	
Lupus	0 (0) *	1 (0.8) *	1 (0.5) *	
Premature	1 (1.1) *	7 (5.6) *	8 (3.7) *	0.142 ^c^
Delivery Type				0.937 ^c^
Normal Birth	50 (100) *	66 (79.5) *	116 (87.2) *	
Cesarean Section	0 (0) *	17 (20.5) *	17 (12.8) *	
Postpartum Stay	1 (1.1) *	12 (9.7) *	13 (6) *	0.019 ^c,1^
Indirect Hyperbilirubinemia	1 (1.1) *	5 (4) *	6 (2.8) *	
Respiratory Distress Syndrome	0 (0) *	7 (5.6) *	7 (3.2) *	
Diagnosis				0.050 ^c^
Acute Bronchiolitis	10 (10.8) *	5 (4) *	15 (6.9) *	0.097
Acute Gastroenteritis	8 (8.6) *	7 (5.6) *	15 (6.9) *	0.562
Bronchopneumonia	0 (0) *	2 (1.6) *	2 (0.9) *	0.508
Urinary Tract Infection	11 (11.8) *	9 (7.3) *	20 (9.2) *	0.360
Acute Otitis Media	15 (16.1) *	16 (12.9) *	31 (14.3) *	0.634
Upper Respiratory Infection	49 (52.7) *	77 (62.1) *	126 (58.1) *	0.165
Temperature (°C)	38.2 (37.9–40.1) ***	38.9 (38–40.9) ***	38.7 (37.9–40.9) ***	<0.001 ^a^

^a^ Mann–Whitney U-test; ^b^ Independent Samples *t*-test; ^c^ Chi-square or Fisher’s Exact test; SD: Standard deviation; *: n (%); **: Mean ± SD (minimum-maximum); ***: Median (interquartile range); SFS: Simple Febrile Seizure; CFS: Complex Febrile Seizure; FS: Febrile Seizure; NA: Not applicable. ^1^ The frequencies of indirect hyperbilirubinemia and respiratory distress syndrome were statistically higher in the patient group.

**Table 3:** Distribution of demographic and clinical findings in patients with simple febrile seizures (SFS) and complex febrile seizures (CFS).

Variables	SFS (n = 86) n (%) or Median (Interquartile Range) or Mean ± SD (Minimum-Maximum)	CFS (n = 38) n (%) or Median (Interquartile Range) or Mean ± SD (Minimum-Maximum)	Total (n = 124) n (%) or Median (Interquartile Range) or Mean ± SD (Minimum-Maximum)	*p*-Value
Age (months)	17 (7–60) **	24 (7–59) **	21.5 (7–60) **	0.141 ^a^
Gender				0.374 ^c^
Male	52 (60.5) *	19 (50) *	71 (57.3) *	
Female	34 (39.5) *	19 (50) *	53 (42.7) *	
Weight SD score	0.3 (−1.4–1.7) **	−0.2 (−1.4–1.2) **	0.2 (−1.4–1.7) **	<0.001 ^a^
Height SD score	0.2 ± 0.06 (−1.3–1.8) ***	−2.2 ± 0.06 (−2.3–0.9) ***	0.1 ± 0.06 (−2.3–1.8) ***	<0.001 ^b^
BMI SD score	0.1 ± 0.4 (−1.8–1.2) ***	−0.1 ± 0.07 (−1.7–3) ***	0.08 ± 0.03 (−1.8–3) ***	0.002 ^b^
Seizure Duration (minutes)	3 (1–10) **	5 (1–20) **	4 (1–20) **	<0.001 ^a^
Family History	48 (55.8) *	35 (92.1) *	83 (66.9) *	<0.001 ^c^
FS (Father)	11 (12.8) *	16 (42.1) *	27 (21.8) *	<0.001 ^c^
FS (Mother)	4 (4.7) *	3 (7.9) *	7 (5.6) *	0.413 ^c^
FS (Brother/Sister)	13 (15.1) *	8 (21.1) *	21 (16.9) *	0.459 ^c^
FS (Uncle)	5 (5.8) *	2 (5.3) *	7 (5.6) *	1.000 ^c^
FS (Cousin)	10 (11.6) *	1 (2.6) *	11 (8.9) *	0.174 ^c^
FS (Aunt)	4 (4.7) *	2 (5.3) *	6 (4.8) *	1.000 ^c^
Epilepsy (Uncle)	2 (2.4) *	1 (2.6) *	3 (2.4) *	1.000 ^c^
Epilepsy (Mother)	1 (1.2) *	3 (7.9) *	4 (3.2) *	0.073 ^c^
Epilepsy (Brother/Sister)	3 (3.5) *	2 (5.3) *	5 (4) *	0.627 ^c^
Epilepsy (Aunt)	0 (0) *	4 (10.5) *	4 (3.2) *	0.006 ^c^
Affinity				0.026 ^c,1^
2nd degree relative	12 (14) *	13 (34.2) *	25 (20.2) *	
3rd degree relative	10 (11.6) *	5 (13.2) *	15 (12.1) *	
Smoking During Pregnancy	30 (34.9) *	16 (42.1) *	46 (37.1) *	0.572 ^c^
Medical Drug Use During Pregnancy	5 (5.8) *	7 (18.4) *	12 (9.7) *	0.045 ^c,2^
Cancer	1 (1.2) *	0 (0) *	1 (0.8) *	
Diabetes Mellitus	1 (1.2) *	5 (13.2) *	6 (4.8) *	
Hypertension	2 (2.4) *	2 (5.3) *	4 (3.2) *	
Systemic Lupus Erythematosus	1 (1.2) *	0 (0) *	1 (0.8) *	
Premature	4 (4.7) *	3 (7.9) *	7 (5.6) *	1.000 ^c^
Delivery Type				0.260 ^c^
Normal Birth	43 (74.1) *	23 (92) *	66 (79.5) *	
Cesarean Section	15 (25.9) *	2 (8) *	17 (20.5) *	
Postpartum Stay	6 (7) *	6 (15.8) *	12 (9.7) *	0.744 ^c^
Indirect Hyperbilirubinemia	2 (2.4) *	3 (7.9) *	5 (4) *	
Respiratory Distress Syndrome	4 (4.7) *	2 (5.3) *	7 (5.6) *	
Diagnosis				0.372 ^c^
Acute Bronchiolitis	3 (3.5) *	2 (5.3) *	5 (4) *	
Acute Gastroenteritis	6 (7) *	1 (2.6) *	7 (5.6) *	
Bronchopneumonia	1 (1.2) *	1 (2.6) *	2 (1.6) *	
Urinary Tract Infection	5 (5.8) *	4 (10.5) *	9 (7.3) *	
Acute Otitis Media	9 (10.5) *	7 (18.4) *	16 (12.9) *	
Upper Respiratory Infection	54 (62.8) *	23 (60.5) *	77 (62.1) *	
Temperature (°C)	38.7 (38–40.9) **	39.9 (38–40.2) **	38.9 (38–40.9) **	<0.001 ^a^

^a^ Mann–Whitney U-test; ^b^ Independent Samples *t*-test; ^c^ Chi-square or Fisher’s Exact test; SD: Standard deviation; *: n (%); **: Median (interquartile range); ***: Mean ± SD (minimum-maximum); SFS: Simple Febrile Seizures; CFS: Complex Febrile Seizures; FS: Febrile Seizures; BMI: Body Mass Index. ^1^ The frequency of 2nd degree relatives has statistically higher in the CFS group; ^2^ The frequency of diabetes mellitus has statistically higher in the CFS group.

**Table 4:** Comparison of laboratory measurements between study and control groups.

Variables	Control (n = 93) Median (Interquartile Range) or Mean ± SD (Minimum-Maximum)	Patient (n = 124) Median (Interquartile Range) or Mean ± SD (Minimum-Maximum)	Total (n = 217) Median (Interquartile Range) or Mean ± SD (Minimum-Maximum)	*p*-Value
Hb (gr/dL)	12.1 ± 0.8 (10–13.9) **	11.2 ± 1.1 (7.8–14.9) **	11.6 ± 1.1 (7.8–14.9) **	<0.001 ^b^
Hct (%)	36.3 ± 2.7 (25.3–42.3) **	34.1 ± 2.8 (26.4–42.4) **	35.0 ± 3.0 (25.3–42.4) **	<0.001 ^b^
WBC (×10^3^/µL)	10.9 (5.3–23.9) *	12.5 (4.6–27.6) *	11.6 (4.6–27.6) *	0.012 ^a^
PLT (×10^3^/µL)	352.5 ± 77.9 (175–510) **	293.1 ± 84.7(132–599) **	318.5 ± 86.8 (132–599) **	<0.001 ^b^
MCV (fL)	79 (62.1–87.4) *	75.3 (52.7–84) *	76.6 (52.7–87.4) *	<0.001 ^a^
Ferritin (ng/mL)	37.6 (3–145.4) *	56.5 (4.9–290.3) *	47.7 (3–290.3) *	<0.001 ^a^
Iron (µg/dL)	68 (13–179) *	43 (9–205) *	51 (9–205) *	<0.001 ^a^
Total Iron Binding Capacity (µg/dL)	305.4 ± 67.2 (176–496) **	357.5 ± 64.2 (206–499) **	335.2 ± 70.3 (176–499) **	<0.001 ^b^
Transferrin saturation	23.1 (3.3–86.3) *	11.5 (2.1–61.9) *	15.7 (2.1–86.3) *	<0.001 ^a^
Prolactin (ng/mL)	14.5 (5.2–41.8) *	27.3 (11.2–71.6) *	19.5 (5.2–71.6) *	<0.001 ^a^
Cortisol (µg/dL)	14.3 (6.4–35.2) *	25.4 (3.4–57.8) *	19.6 (3.4–57.8) *	<0.001 ^a^
Vitamin B12 (pg/mL)	486.1 (199.5–1196) *	442 (140.7–1039) *	454.1 (140.7–1196) *	0.005 ^a^
Folate (ng/mL)	8.9 (4–20) *	9.7 (2.1–20) *	9.3 (2.1–20) *	0.521 ^a^
Vitamin D (ng/mL)	27.8 (8.1–53) *	16 (3.3–67.5) *	21.2 (3.3–67.5) *	<0.001 ^a^
FT4 (ng/mL)	1.3 (0.8–1.9) *	1.3 (0.9–1.7) *	1.3 (0.8–1.9) *	0.974 ^a^
TSH (µIU/mL)	2.1 (0.2–5.4) *	2.3 (0.6–5.7) *	2.3 (0.2–5.7) *	0.282 ^a^
Sodium (mmol/L)	138 (130–144) *	133 (121–140) *	136 (121–144) *	<0.001 ^a^
Potassium (mmol/L)	4.4 (3–5.5) *	4.3 (3.4–5.4) *	4.3 (3–5.5) *	0.011 ^a^
Chlorine (mmol/L)	102 (91–109) *	103 (95–111) *	102 (91–111) *	0.127 ^a^
Glucose (mg/dL)	97 (75–168) *	131 (80–242) *	110 (75–242) *	<0.001 ^a^
AST (U/L)	30 (11–57) *	31 (19–105) *	30.9 (11–105) *	0.101 ^a^
ALT (U/L)	15 (6–115) *	15 (2–85) *	15 (2–115) *	0.310 ^a^
Calcium (mg/dL)	9.8 (8.3–11)	9.5 (8.5–10.8)	9.6 (8.3–11)	<0.001 ^a^
Urea (mg/dL)	22 (11–42)	22 (6–47)	22 (6–47)	0.566 ^a^
Creatinine (mg/dL)	0.4 (0.2–0.8) *	0.4 (0.2–0.6) *	0.4 (0.2–0.8) *	0.595 ^a^
CRP (mg/L)	11.3 (1.2–70.2) *	11.8 (0.8–115.7) *	11.5 (0.8–115.7) *	0.909 ^a^
Magnesium (mg/dL)	2 (1.7–2.4) *	2 (1.7–2.8) *	2 (1.7–2.8) *	0.873 ^a^
Phosphorus (mg/dL)	4.9 (3.5–6.4) *	4.8 (3–6.4) *	4.8 (3–6.4) *	0.200 ^a^
Zinc (µg/dL)	110 (55–238) *	69 (33–222) *	89 (33–238) *	<0.001 ^a^
Selenium (µg/L)	0 *** (0)	49 (21.9–161)	44.9 (21.9–161)	NA ***

^a^ Mann–Whitney U-test; ^b^ Independent Samples *t*-test; SD: Standard deviation; * Median (interquartile range); ** Mean ± SD (minimum-maximum); *** NA: Not applicable; Hb: Hemoglobin; Hct: Hematocrit; WBC: White Blood Cell count; PLT: Platelet count; MCV: Mean Corpuscular Volume; FT4: Free Thyroxine; TSH: Thyroid Stimulating Hormone; AST: Aspartate Aminotransferase; ALT: Alanine Aminotransferase; CRP: C-Reactive Protein.

**Table 5:** Distribution of laboratory measurements in patients with simple febrile seizures (SFS) and complex febrile seizures (CFS).

Variables	SFS (n = 86) Median (Interquartile Range) or Mean ± SD (Minimum-Maximum)	CFS (n = 38) Median (Interquartile Range) or Mean ± SD (Minimum-Maximum)	Total (n = 124) Median (Interquartile Range) or Mean ± SD (Minimum-Maximum)	*p*-Value
Hb (gr/dL)	11.4 ± 1.0 (9–14.9) **	10.9 ± 1.2 (7.8–13.9) **	11.2 ± 1.1(7.8–14.9) **	0.042 ^b^
Hct (%)	34.5± 2.5(29.2–42.4) **	33.1 ± 3.2 (26.4–40.2) **	34.1 ± 2.8 (26.4–42.4) **	0.034 ^b^
WBC (×10^3^/µL)	11.2 (4.6–25.8) *	15.9 (5.7–27.6) *	12.5 (4.6–27.6) *	<0.001 ^a^
PLT (×10^3^/µL)	314.5 ± 84.0 (140–599) **	244.8 ± 64.6 (132–516) **	293.1 ± 84.7 (132–599) **	<0.001 ^b^
MCV (fL)	75.6 (52.7–84) *	74.6 (56.7–82.8) *	75.3 (52.7–84) *	0.156 ^a^
Ferritin (ng/mL)	46.5 (4.9–189.6) *	108.6 (7.8–290.3) *	56.5 (4.9–290.3) *	<0.001 ^a^
Iron (µg/dL)	50.5 (12–205) *	29.5 (9–92) *	43 (9–205) *	<0.001 ^a^
Total iron binding capacity (µg/dL)	338.3 ± 56.1 (206–461) **	400.8 ± 60.9 (252–499) **	357.5 ± 64.2 (206–499) **	<0.001 ^b^
Transferrin saturation (%)	14.7 (3.6–61.9) *	7.8 (2.1–22.4) *	11.5 (2.1–61.9) *	<0.001 ^a^
Prolactin (ng/mL)	20.8 (11.2–53.3) *	43.4 (25.1–71.6) *	27.3 (11.2–71.6) *	<0.001 ^a^
Cortisol (µg/dL)	20.6 (3.4–44.1) *	37.4 (10.8–57.8) *	25.4 (3.4–57.8) *	<0.001 ^a^
Vitamin B12 (pg/mL)	444.1 (140.7–1039) *	433.6 (196.9–751.1) *	442 (140.7–1039) *	0.610 ^a^
Folate (ng/mL)	9.9 (4.4–20) *	9.2 (2.1–19.3) *	9.7 (2.1–20) *	0.443 ^a^
Vitamin D (ng/mL)	20.6 (5.2–67.5) *	9.8 (3.3–18.6) *	16 (3.3–67.5) *	<0.001 ^a^
T4 (ng/mL)	1.3 (1–1.7) *	1.3 (0.9–1.7) *	1.3 (0.9–1.7) *	0.566 ^a^
TSH (µIU/mL)	2.3 (0.7–5.7) *	2.3 (0.6–5.7) *	2.3 (0.6–5.7) *	0.931 ^a^
Sodium (mmol/L)	134 (121–140) *	130 (124–133) *	133 (121–140) *	<0.001 ^a^
Potassium (mmol/L)	4.3 (3.4–5.4) *	4.2 (3.4–5.3) *	4.3 (3.4–5.4) *	0.420 ^a^
Chlorine (mmol/L)	103 (96–111) *	101 (95–110) *	103 (95–111) *	0.040 ^a^
Glucose (mg/dL)	118 (80–242) *	175 (127–231) *	131 (80–242) *	<0.001 ^a^
AST (U/L)	31.5 (19–105) *	30 (19–54) *	31 (19–105) *	0.147 ^a^
ALT (U/L)	14 (2–85) *	16.9 (8–36) *	15 (2–85) *	0.085 ^a^
Calcium (mg/dL)	9.4 (8.5–10.8) *	9.6 (8.8–10.5) *	9.5 (8.5–10.8) *	0.698 ^a^
Urea (mg/dL)	20.5 (6–47) *	24 (13–44) *	22 (6–47) *	0.013 ^a^
Creatinine (mg/dL)	0.4 (0.2–0.6) *	0.4 (0.2–0.6) *	0.4 (0.2–0.6) *	0.200 ^a^
CRP (mg/L)	8 (0.8–115.7) *	21.1 (5.1–92.1) *	11.8 (0.8–115.7) *	<0.001 ^a^
Magnesium (mg/dL)	2.1 (1.8–2.8) *	2 (1.7–2.4) *	2 (1.7–2.8) *	0.081 ^a^
Phosphorus (mg/dL)	4.8 (3–6.4) *	4.6 (3.1–5.7) *	4.8 (3–6.4) *	0.118 ^a^
Zinc (µg/dL)	84.4 (40–222) *	57.1 (33–155) *	69 (33–222) *	<0.001 ^a^
Selenium (µg/L)	49 (21.9–161) *	34.7 (24.4–73) *	49 (21.9–161) *	0.019 ^a^

^a^ Mann–Whitney U-test; ^b^ Independent Samples *t*-test; SD: Standard deviation; *: Median (interquartile range); ** Mean ± SD (minimum-maximum); Hb: Hemoglobin; Hct: Hematocrit; WBC: White Blood Cell count; PLT: Platelet count; MCV: Mean Corpuscular Volume; FT4: Free Thyroxine; TSH: Thyroid Stimulating Hormone; AST: Aspartate Aminotransferase; ALT: Alanine Aminotransferase; CRP: C-Reactive Protein.

**Table 6:** Comparison of laboratory parameters by number of seizures.

Variables	<2 Seizures Median (Interquartile Range)	≥2 Seizures Median (Interquartile Range)	*p*-Value
Iron (µg/dL)	49 (12–205)	42 (9–119)	0.158 ^a^
Vitamin B12 (pg/mL)	445.2 (224.1–937.2)	441.1 (140.7–1039)	0.239 ^a^
Folate (ng/mL)	10.3 (4.4–20)	9.5 (2.1–20)	0.629 ^a^
Vitamin D (ng/mL)	21.2 (5–57.4)	13.3 (3.3–67.5)	<0.001 ^a^
Zinc (µg/dL)	89 (54–222)	60.2 (33–167)	<0.001 ^a^
Selenium (µg/L)	53.1 (26.2–112)	39 (21.9–161)	<0.001 ^a^

^a^ Mann–Whitney U-test.

**Table 7:** Genetic variants in patients with febrile seizures: genetic and clinical findings.

n	S	Age	ST	Gene	Trancript	Nucleotide Change	Amino Acid Change	Exon	ACMG	I
1	M	25	SFS	AIMP1	ENST00000394701.4	c.263_264del	p.Q88Rls*25	31	VUS, Novel	AR
2	M	44	CFS	AP4B1	NM_001253852.3	c.1497G>T	p.Leu499Phe	5	LB	AR
3	M	21	CFS	ASPM	NM_018136.5	c.5430_5431insAGTA	p.A1811Sfs*7	18	LP, Novel	AR
4	M	23	SFS	BTD	NM_001281723.3	c.565C>T	p.R189C	4	P	AR
5	M	13	SFS	CACNA1A	NM_023035.3	c.4460G>A	p.R1487H	28	LP	AD
6	F	8	CFS	CHD2	NM_001271.4	c.3942G>A	p.A1314	31	LB	AD
7	M	25	CFS	CHD2	NM_001271.4	c.2636C>T	p.Ala879Val		LP	AD
8	F	13	CFS	CLN6	NM_017882.3	c.755G>T	p.R252L	7	VUS, Novel	AR
9	M	25	CFS	CNTNAP2	NM_014141.6	c.1855A>T	p.5619C	12	VUS	AR
10	M	10	CFS	CNTNAP2	NM_014141.6	c.3344T>C	p.Val1115Ala		VUS	AR
11	M	18	SFS	GABRG2	NM_198903.2	c.*1427C>T	3UTR		Benign	AD
12	F	15	SFS	GOSR2	ENST0000225567.4	c.109A>T	p.137F		VUS	AR
13	M	28	CFS	IL6	NM_000600.5	c.155G>T	pArg52Leu	2	VUS	AD/AR
14	M	25	SFS	KCNJ1	ENST00000392664.2	c.658C>T	p.L220F	2	LP	AR
15	M	22	SFS	KCNQ3	NM_004519.4	C.5580G>A	3UTR		VUS	AD
16	M	18	SFS	KCNT1	ENST00000371757.2	c.2062G>A	p.G6885	19	VUS	AD
17	F	36	CFS	LDRL	NM_00527.5	c.2475C>A	N825K	17	LP	AD/AR
18	F	13	SFS	LGI1	ENST00000371418.4	c.1580A>G	p.H527R	8	VUS	AD
19	M	27	SFS	MAGI2	NM_012301.4	c.1880G>A	p.R627Q		VUS	AR
20	F	29	CFS	MFSD8	NM_152778.3	c.*463A>T	3UTR		VUS	AR
21	M	60	CFS	PCDH19	NM_001184880.2	c.369C>G	p.N123K rs796052796	1	LP	XL RES
22	F	10	CFS	PCDH19	NM_001184880.2	c.1707del T	p.Asn570Thrfs		P, Novel	
23	F	18	CFS	PCDH19	NM_001184880.2	c.34_36delCTG	p.Leu12del		LB	
24	F	14	CFS	PCDH19	NM_001184880.2	c.1142A>G	p.Asn381Ser		LP	
25	F	7	CFS	PLA2G6	ENST00000427453.1	c.355C>T	p.Q119	4	LP	AR
26	M	13	SFS	POLG	NM_001126131.2	c.1910G>A	p.G637D	10	LB	AD/AR
27	M	7	SFS	SCN1A	NM_001165963.3	c.*1872del	3UTR		VUS	AD
28	M	16	SFS	SCN1A	NM_001165963.3	c.2792G>A	p.R931H	8	P	AD
29	F	18	CFS	SCN1A	NM_001165963.4	c.4681G>A	p.Glu1561Lys		LP	
30	F	7	SFS	SCN1A	NM_001165963.2	c.4933C>G	p.Arg1645Gly	26	P	AD
31	F	6	SFS	SCN1A	NM_001165963.4	c.4933C>G	p.R1645G	29	P	AD
32	M	16	SFS	SCN1A	NM_001165963.4	c.3327dup	p.S1110Qfs*14		P	
33	M	32	CFS	SCN2A	NM_001040142.2	c1819C>t	p.Arg607	12	P	AD
34	F	36	CFS	SCN9A		c.5790_5797dupAGATATGG	p.Ala1933fs rs746685972	27	P, novel	AR
35	M	25	CFS	SLC13A5	NM_177550.5	c.1366G>A	p.V4561	10	LB	AR
36	F	21	CFS	TBCD24	NM_001199107.2	c.*1658C>T	3UTR		LB	AD/AR
37	F	29	CFS	ZEB2	NM_014795.4	C.*1932G>T	3UTR		LB	AD
38	F	19	CFS	GRIN2A		c.3221C>T	p.Pro1074Leurs867432846	14	LB	AD

n: Number of patients; S: Sex; M: Male; F: Female; Age: Month; ST: Seizure type; ACMG: American College of Medical Genetics classification; SFS: Simple Febrile Seizure; CFS: Complex Febrile Seizure; P: Pathogenic; LP: Likely Pathogenic; LB: Likely Benign; VUS: Variant of Uncertain Significance; Het: Heterozygous; AD: Autosomal Dominant; AR: Autosomal Recessive; XL RES: X-linked Recessive; I: Inheritance.

**Table 8:** Comparison of laboratory parameters based on genetic results in patients with febrile seizures.

Variables	Genetic Negative Median (Interquartile Range)	Genetic Positive Median (Interquartile Range)	*p*-Value
Iron (µg/dL)	49 (12–205)	37.5 (9–111)	0.029 ^a^
Vitamin B12 (pg/mL)	444.1 (214.9–1039)	439.6 (140.7–751.1)	0.560 ^a^
Folate (ng/mL)	9.6 (2.1–20)	10 (2.6–20)	0.933 ^a^
Vitamin D (ng/mL)	19.7 (3.3–67.5)	11.8 (4.9–50.1)	<0.001 ^a^
Zinc (µg/dL)	89 (39–222)	55.4 (33–155)	<0.001 ^a^
Selenium (µg/L)	53.1 (29.9–161)	29.2 (21.9–55.2)	<0.001 ^a^

^a^ Mann–Whitney U-test.

## Data Availability

All data supporting the findings of this study are available within the paper.
